# Magnetocaloric Properties and Microstructures of HoB_2_ and Nb-Substituted HoB_2_

**DOI:** 10.3390/ma18040866

**Published:** 2025-02-17

**Authors:** Mahboobeh Shahbazi, Ali Dehghan Manshadi, Kiran Shinde, Ian D. R. Mackinnon

**Affiliations:** 1 Centre for Materials Science and School of Chemistry and Physics, Queensland University of Technology, Brisbane, QLD 4001, Australia; mahboobeh.shahbazi@qut.edu.au; 2 Centre for Clean Energy Technologies and Practices, Queensland University of Technology, Brisbane, QLD 4001, Australia; 3 School of Mechanical and Mining Engineering, The University of Queensland, Brisbane, QLD 4072, Australia; aliuow@gmail.com; 4 Department of Nanotechnology and Advanced Materials Engineering, Sejong University, Seoul 05006, Republic of Korea; yourkirans@gmail.com; 5 School of Earth and Atmospheric Sciences, Queensland University of Technology, Brisbane, QLD 4001, Australia

**Keywords:** magnetocaloric materials, Holmium diboride, niobium, microstructure, Curie temperature, magnetic entropy

## Abstract

We report on the arc melt syntheses of HoB_2_ and Nb-substituted HoB_2_ polycrystalline ingots and their magnetocaloric and microstructural properties. XRD data and microstructural analysis reveal that a nominal 10% Nb addition during synthesis results in changes to unit cell parameters and grain morphology. Interpretation of the refined cell parameters using Vegard’s law shows that Nb substitutes into HoB_2_ with stoichiometry Ho_0.93_Nb_0.07_B_2_. Arc-melted products are polycrystalline bulk samples containing minor phases such as Ho_2_O_3_, Ho, and HoB_4_. Nb substitution results in a smaller grain size (~sub-micron) and a higher Curie temperature, T_C_, compared to HoB_2_. With a 10 T applied field, the maximum magnetic entropy, ΔS_M_, for HoB_2_ and for Ho_0.93_Nb_0.07_B_2_, is 46.8 Jkg^−1^K^−1^ and 38.2 Jkg^−1^K^−1^ at 18 K and 21 K, respectively. Both samples show second-order phase transitions. Despite high totals of minor phases (e.g., ~10 wt.% and ~25 wt.%), the calculated relative cooling powers are greater than 1300 Jkg^−1^ and 600 Jkg^−1^ at 10 T and 5 T, respectively. The magnetocaloric properties of both samples are consistent with Holmium boride compounds prepared via alternative methods.

## 1. Introduction

The global demand for reduced CO_2_ emissions has increased the attention paid to the use of renewable energy, including green hydrogen as an energy vector. Energy storage is a key issue to be addressed for the widespread adoption of renewable energy in domestic or export markets [1,2,3]. In this regard, liquid hydrogen is a likely medium for storing, transporting, and using renewable energy [4] over a wide range of scales and applications [5]. Liquefaction methods for hydrogen typically involve a combination of compression, expansion, and throttling processes, such as with the Linde–Hampson cycle [5,6]. Currently, on an industrial scale, the largest single liquefier has a capacity of 32 T/day [5]. In these cases, cyclic gas compression techniques are the predominant cooling methods, and contribute to the high operational and capital costs of many installations [5,7].

Recent attention has been directed at magnetic refrigeration (MR) methods, with a focus on small- to modest-scale refrigeration techniques, as succinctly described in a review article by Kitanovski [8]. Kitanovski [8] suggests that the low Carnot efficiency of existing refrigeration at a small scale offers substantial room for improvement by using MR technologies. An exemplar use of MR is described by Archipley et al. [9], who use active magnetic regenerative refrigeration (AMRR) to liquefy methane at room temperature.

Magnetocaloric (MC) materials show properties that invoke an isothermal magnetic entropy change or an adiabatic temperature change with the application or removal of an external magnetic field. This phenomenon is known as a magnetocaloric effect (MCE), with key applications in MR. The use of MCE has been demonstrated for room temperature applications with compounds such as Gd_5_Si_2_Ge_2_, (Mn,Fe)_2_P, La(Fe,Si)_13_H, and Gd_0.8875_Ce_0.1025_Si_0.84_Cr_0.19_ [10,11,12,13,14]. MR research has also focused on cryogenic temperatures [15,16,17,18,19], especially for hydrogen liquefaction, which occurs at 20 K at atmospheric pressure. A report by Ihnfeldt et al. [20] shows that an MR system that achieves 50% Carnot efficiency in the 20–80K region would provide an 85% reduction in electrical costs and a 60% reduction in the capital equipment cost compared to traditional compression-based cryocoolers.

Bykov et al. [17] and Tang et al. [19] have shown that combinations of stacked MC materials are suitable for cooling from 77 K to liquid hydrogen temperature. Material combinations include first-order (FOPT) and second-order phase transition (SOPT) MCE compounds such as ErCo_2_ and HoB_2_ [19], or tuning the Curie temperature, T_C_, with various substituent elements (e.g., Ho_1−x_Dy_x_Al_2_ or Ho_1−x_Gd_x_B_2_) [17,21]. A giant MCE reported for the rare-earth diboride, HoB_2_, shows strong potential for low-temperature applications at or near the Curie temperature, T_C_, of ~15 K [22]. The maximum change in magnetic entropy, ΔS_M_, for HoB_2_ at 5 T is 40.1 Jkg^−1^K^−1^ [22], and is of optimum practical use near 15 K. Other Ho-based compounds such as HoAl_2_ and Ho_1-x_Gd_x_B_2_ show maximum ΔS_M_ values above and below 20 K at 5 T [17,21].

In this work, we consider combinations of Ho-based compounds that may be suitable for MR at temperatures <77 K. For hydrogen liquefaction plants on an industrial scale, superconducting magnets are considered a highly effective choice [17,18], not only for large-scale production [17], but also because fields of 10 T or higher are attainable. We explore applied magnetic fields up to 10 T combined with an increase in T_C_ via compatible element substitution into HoB_2_. In this study, we report on Nb substitution in the Ho-B alloy system and the effects on the microstructure and magnetocaloric properties.

## 2. Materials and Methods

Polycrystalline samples of Ho-B compounds are prepared on a water-cooled copper hearth via arc melting using a tungsten electrode, and high-purity Ar. Stochiometric amounts of Ho (95% purity, supplied by Sigma Aldrich, Ryde, NSW, Australia) and nano boron powder (99.8% purity, supplied by Pavezynum Co., Gebze, Kocaeli, Turkey) are weighed and pressed into a pellet of a weight of 2 g. Details of the impurities detected in the Ho powder using ICP-OES analysis are provided in Supplementary Materials Table S1. For substituted samples, molar ratios of Ho, B, and Nb are also weighed (for nominal 10% of a substituent element), mixed, and pressed into a pellet of a weight of 2 g. Both pellets are formed under an applied pressure of 10 tonnes for 2 min.

The pellets are then melted in an arc furnace on a water-cooled copper hearth under Ar. The use of a water-cooled hearth with the arc melting technique helps to reduce contamination from the crucible that contains the starting mixture, and allows for the easy removal of oxygen from the surrounding gas in the arc furnace. Prior to arc melting experiments, the chamber is vented and filled with Ar three times, and then re-filled with Ar in order to eliminate oxygen in the chamber. The Ar atmosphere is further purified using Ti foam before melting each pellet. In order to ensure element homogeneity in the mixed material, the ingot is turned and remelted four times utilizing the same heating and cooling rates for each sample.

The microstructural and compositional analyses were performed using standard metallographic practices on polished samples mounted in conductive resin. The crystal structure and phase identification were analyzed using X-ray diffraction (XRD), scanning electron microscopy (SEM), and EDS microanalysis using secondary X-rays. The XRD patterns of samples were measured using Co Kα1 radiation in Bragg–Brentano geometry, with 2θ steps of 0.02° and a counting time of 10 s per step, utilizing D8 Bruker X-ray diffractometers (Bruker, Billerica, MA, USA). The diffraction patterns are refined and indexed using the software programme Topas [23]. Detailed analyses using XRD patterns and SEM+EDS indicate that all synthesized samples are multiphase, with HoB_2_ being the predominant phase.

Electron Backscatter Diffraction (EBSD) data were obtained using a field emission scanning electron microscope (FESEM, JEOL 7001 SEM, Japan Electron Optics Ltd., Tokyo, Japan) with automated feature detection and equipped with an SDD XMax 50 mm^2^ detector, pattern analyzer, and Channel 5 analysis software (Oxford Instruments plc, Abingdon, Oxfordshire, UK). EBSD mapping was conducted at an accelerating voltage of 20 kV and a step size of 0.2 µm.

The temperature and field dependence of dc magnetization measurements were calculated using a Dynacool Physical Property Measurement System (PPMS) with a Vibrating Sample Magnetometer (VSM) from Quantum Design (San Diego, CA USA), in the temperature range of 5–71 K and a dc magnetic field from 0 T to 10 T. The temperature dependence of magnetization (M(T)) was measured under the zero field cooled (ZFC) and field cooled (FC) protocols. The magnetic entropy change is calculated from the isothermal field dependent magnetization curves using Maxwell’s relation [24]:

(1) $$∆S_{m}\left(T,H\right)=\mu_{0\;}\int_{H_{i}}^{H_{f}}(\frac{\partial M}{\partial T}{)}_{H^{'}}dH^{'}\;$$

where *H_i_* is the initial magnetic field and *H_f_* is the final magnetic field.

## 3. Results

The summary details of the physical and chemical properties and respective magnetic properties of HoB_2_ and Nb-substituted HoB_2_ are provided below.

### 3.1. Structural and Microstructural Analysis

Table 1 lists the starting materials, ratios, and proportions of synthesized products using data from XRD measurements. Phase analyses using Rietveld refinements of the XRD data show that HoB_2_ is the major phase, with a maximum yield of 92%. A minor amount of unreacted Ho (3.9%) is also present along with Ho_2_O_3_ (4.0%), despite processing being explicitly aimed at minimizing the potential for oxidation. Table 1 shows that Nb addition reduces the proportion of HoB_2_—in this case, with nominal 10% Nb addition—to 72.1%. Minor proportions of HoB_4_, HoB_12_, and NbB_2_ (less than 10% of each) are detected with the addition of Nb (Table 1).

**Figure 1:**
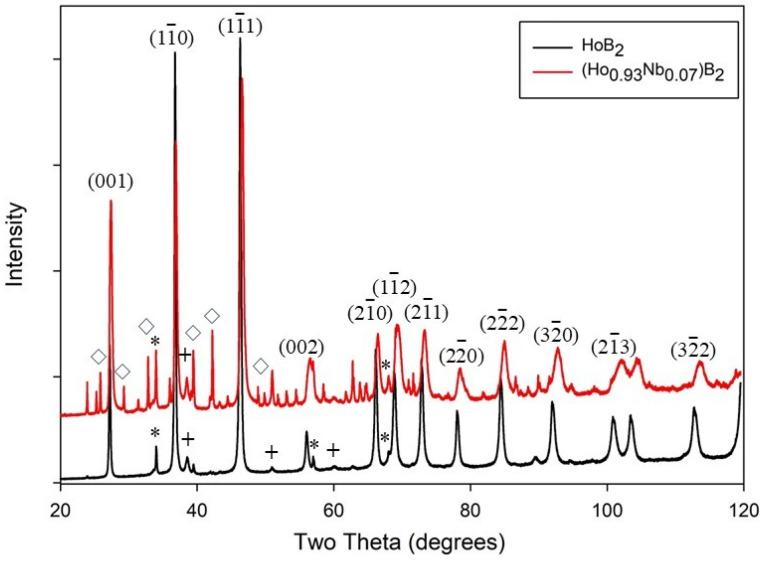
Powder XRD patterns for HoB_2_ (black) and Ho_0.93_Nb_0.07_B_2_ (red) produced via arc melting, as listed in Table 1. Peaks for the HoB_2_ structure are indexed. Peaks for minor impurity phases are identified as + Ho; * Ho_2_O_3_; and ◊ HoB_4_.

Figure 1 shows the XRD patterns for the products listed in Table 1. The peaks are well matched to and indexed for HoB_2_ based on space group P6/mmm and previously determined cell parameters using the powder diffraction file PDF# 04-003-0232. Trace amounts of unreacted Ho and Ho_2_O_3_ (<5%) are also present for synthesis without Nb addition, as noted in Table 1. For both samples, the powder diffraction file (PDF) data are used to identify the presence of other minor phases such as HoB_4_, Ho_2_O_3_, and NbB_2_. Figure 1 shows the XRD data for both samples, with minor impurities identified and indexed peaks for HoB_2_ and Ho_1−x_Nb_x_B_2_.

With the diffraction peaks and minor phases identified, the unit cell parameters for the primary phase, HoB_2_, were refined to a = 3.28296(2) Å and c = 3.814454(4) Å. The refinement of the unit cell parameters for the sample with Nb in the synthesis resulted in shifts in the values to a = 3.268009(6) Å and c = 3.784152(1) Å, as shown in Table 2. These changes in cell parameters are reflected in the clear shift in the (1-10) and (1-11) peaks to higher two-theta angles with Nb addition, as shown in Figure 1. Additional plots of the XRD data, for the intervals 30° < 2θ < 50° and 60° < 2θ < 80°, highlighting this shift to a higher 2θ with Nb substitution, are shown in Figures S1 and S2 of the Supplementary Materials. Figure S1 clearly shows the shift to higher 2θ values for the peaks and Figure S2 highlights the peak broadening, commonly associated with a smaller grain size (compared to HoB_2_). These data are consistent with the substitution of Nb into HoB_2_ and microstructural changes, as confirmed using Vegard’s law.

The methods to establish the level of substitution of soluble elements into metals or minerals of a known structure include single crystal and powder X-ray or neutron diffraction [26,27]. Vegard’s law [28,29] attributes the linear relationship of end-member unit cell parameters to the mixing of components in a substitutional solid solution, particularly for metals and alloys of similar structure. Supplemental Figure S3 shows a plot of a and c cell parameters for the end members HoB_2_ and NbB_2_, as well as for the (Ho,Nb)B_2_ sample prepared in this work. The cell parameter values used for HoB_2_ and NbB_2_ are as shown in Table 2. We conclude from Figure S3 that Nb is soluble in HoB_2_, and is less than the nominal 10% proportion used during synthesis. Using the Vegard plot (Figure S3), we estimate that the relative percentages of Ho and Nb are 92.6(6) and 7.4(6), respectively. For convenience, we show the stoichiometry as (Ho_0.93_Nb_0.07_)B_2_.

**Figure 2:**
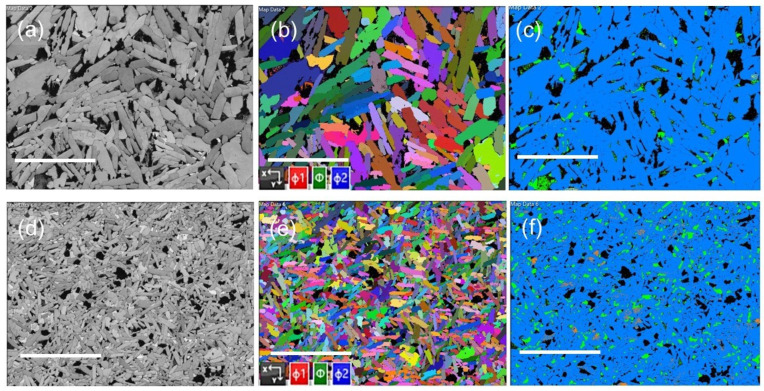
Micrographs of HoB_2_ (top row) and Ho_0.93_Nb_0.0.07_B_2_, (bottom row) showing (**a**,**d**) BSE images of aggregates in polished sections, (**b**,**e**) Euler maps of these aggregates showing the preferred polar orientation of grains for HoB_2_ and Ho_0.93_Nb_0.0.07_B_2_, respectively. (**c,f**) EBSD images for aggregates from the same areas in (**b**,**e**) showing HoB_2_ (blue), Ho_2_O_3_ (yellow), Ho (green), NbB_2_ (orange), and void (black). Note the substantial difference in grain size for each sample. All images are at the same magnification (white scale bar = 50 µm).

Backscattered electron (BSE) images from polished samples of HoB_2_ and Ho_0.93_Nb_0.07_B_2_ are shown in Figure 2a and Figure 2d, respectively. BSE images show that aggregates contain different phases, as indicated by the bright and dark grey image contrast, which typically corresponds to variations in atomic number. In general, these images are consistent with the phase abundances obtained by Rietveld refinement of the XRD data shown in Table 1. The small white spots in the BSE images correspond to Ho_2_O_3_. Compared to HoB_2_, polished samples for Ho_0.93_Nb_0.07_B_2_ show fewer voids.

The Euler maps and EBSD images in Figure 2 show the crystal orientation and structure of component alloys using known crystallographic data for HoB_2_, HoB_4_, Ho, Ho_2_O_3_, and NbB_2_. EBSD analyses without the use of noise reduction software applied to the maps are presented in Figure 2b,e. Figure 2c,f show that HoB_2_ is a major phase for both samples (blue-coloured grains) and confirm the XRD data shown in Table 1. Ho_2_O_3_ is present in all samples (indicated by yellow grains in Figure 2c,f), although the size and relative abundance of the oxide varies between samples. Figure 2d–f show that grains produced via mixing Nb with Ho and B during arc melting are significantly smaller than those in HoB_2_.

### 3.2. Magnetocaloric Properties

**Figure 3:**
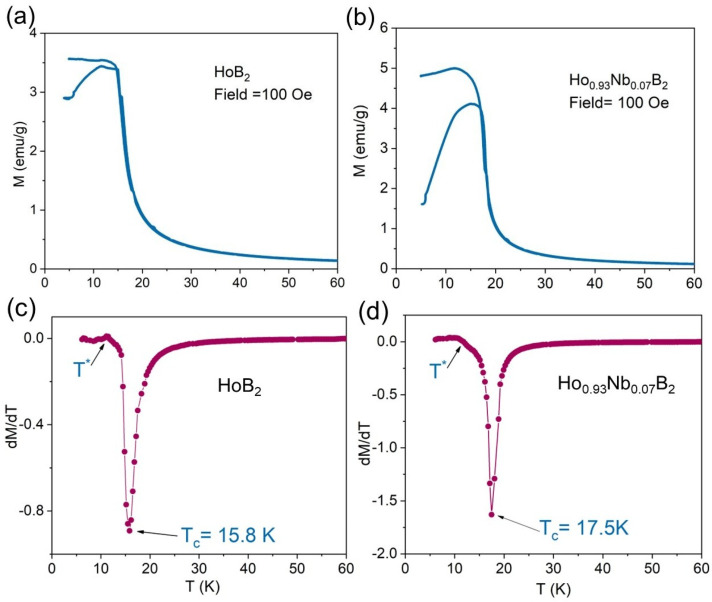
Magnetic properties of HoB_2_ (left panel) and Ho_0.93_Nb_0.0.07_B_2_ (right panel) showing (**a**,**b**) temperature dependence of sample magnetization under an applied magnetic field of 0.01 T using zero field cooling (ZFC) protocols and (**c**,**d**) the derivative of ZFC protocols to denote temperature dependence of ∂M∂T. A small kink anomaly associated with a spin orientation phenomenon identified as T* is arrowed.

Figure 3a,b show the temperature dependence of magnetization (M-T) for both synthesized alloys under an applied field of 100 Oe. For Ho_0.93_Nb_0.07_B_2_, the divergence between the zero field cooled (ZFC) and field cooled (FC) M-T curves is more pronounced than for HoB_2_. For ZFC conditions, the magnetization slowly increases and then rises sharply between 15 and 18 K, exhibiting typical paramagnetic to ferromagnetic transitions. To evaluate the magnetic transition temperature, T_C_, the temperature dependent derivatives of the FC curves are shown in Figure 3c,d. The T_C_ is defined as the minimum in dM/dT, and is 15.8 K for HoB_2_. This value for T_C_ is similar to that reported by de Castro et al. [22] (15 K) for arc-melted HoB_2_. For Ho_0.93_Nb_0.07_B_2_, Figure 3d shows an increase in T_C_ to 17.5 K. All the δM-δT curves exhibit a kink anomaly around 11 K (T^*^), which is associated with a spin reorientation phenomenon [30].

**Figure 4:**
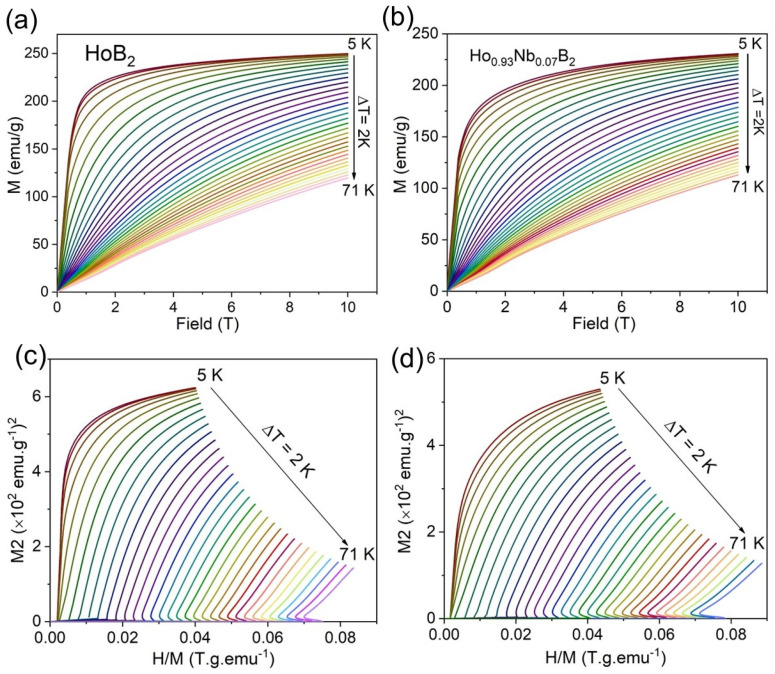
Field dependence of magnetization for (**a**) HoB_2_ and (**b**) Ho_0.93_Nb_0.07_B_2_ at several temperatures below and above the Curie temperature. Arrott plots for (**c**) HoB_2_ and (**d**) (Ho_0.93_Nb_0.07_)B_2_ are obtained from the isothermal magnetization curves of Figure 4a and Figure 4b, respectively.

Figure 4a,b show isothermal magnetization curves measured to the maximum magnetic field of 10 T around T_C_, using a temperature difference interval of 2 K. Magnetization increases rapidly with the increasing magnetic field for temperatures below the Curie temperature, T_C_, for both samples, and tends to be saturated above 5 T. This response is typical ferromagnetic behaviour for intermetallic alloys. For temperatures above T_C_, for example, at 20 K, the magnetization increases almost linearly with the increasing magnetic field. This linear behaviour indicates that these Ho diboride samples are paramagnetic above the Curie temperature.

A plot of H/M versus M^2^, known as the standard Arrott plot, is shown in Figure 4c,d for HoB_2_ and Ho_0.93_Nb_0.07_B_2_, respectively. According to the criterion proposed by Banerjee [31], the order of magnetic field can be determined from the slope of the isothermal plot. If the H/M vs M^2^ curve shows a negative slope, the transition is first order (FOPT), while a positive slope corresponds to a second-order phase transition (SOPT). For both HoB_2_ and Ho_0.93_Nb_0.07_B_2_, neither a negative slope nor an inflexion can be observed in these Arrott plots. Thus, these Ho diboride samples show a SOPT in good agreement with the study on Gd-substituted HoB_2_ [21].

**Figure 5:**
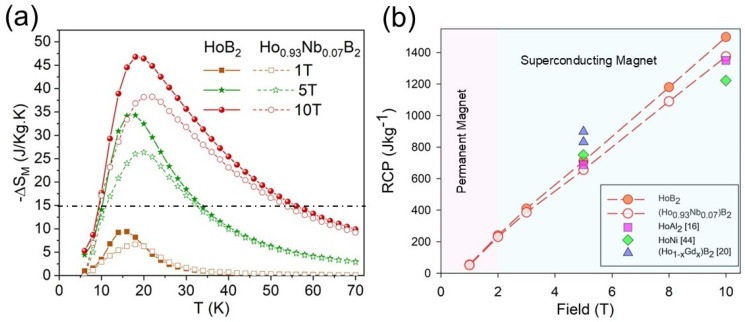
(**a**) Magnetic entropy change, ΔS_M_, for selected applied magnetic fields up to ΔH = 10 T; horizontal dotted line represents a minimum value for a viable MCE at low temperatures [22]. Filled symbols are HoB_2_ and unfilled symbols are Ho_0.93_Nb_0.07_B_2_ at 1 T (squares), 5 T (stars) and 10 T (circles), respectively. (**b**) Field dependence of RCP for HoB_2_ (filled circle) and Ho_0.93_Nb_0.07_B_2_ (unfilled circle) for applied magnetic fields up to 10 T, as well as other Ho compounds at 5 T and 10 T (HoAl_2_: square; HoNi: diamond; (Ho_1-x_Gd_x_)B_2_: triangle). The pink colour denotes operating region for permanent magnets and green denotes additional operating region for superconducting magnets.

Figure 5a shows the calculated magnetic entropy change, ΔS_M_, for applied magnetic fields, ΔH, up to 10 T, with changes in temperature for HoB_2_ and Ho_0.93_Nb_0.07_B_2_. For magnetic fields between 1 and 10 T, the maximum value of magnetic entropy change is 46.8 Jkg^−^^1^K^−^^1^ for HoB_2_ at 10 T and 18 K. Note that the maximum value for ΔS_M_ occurs at temperature(s) slightly higher than the T_C_ value (e.g., 15.8 K for HoB_2_ in this study).

The maximum ΔS_M_ decreases to 38.2 Jkg^−^^1^K^−^^1^ for Ho_0.93_Nb_0_._07_B_2_ at 10 T and 21 K. This reduction in the ΔS_M_ value with element substitution is in good agreement with the earlier work by de Castro et al. [21,32] on the substitution of Gd and Dy in HoB_2_. The reduction in ΔS_M_ with increased T_C_ for Nb-substituted HoB_2_ is also consistent with the first rule proposed by Liu et al. [33], which states that the maximum magnetic entropy change in a rare-earth-based intermetallic series increases as the Curie temperature decreases. The horizontal dotted line in Figure 5a represents a minimum value for ΔS_M_, considered a viable MCE for MR utilized by de Castro et al. [22] for the machine learning discovery of HoB_2_.

The relative cooling power (RCP) of magnetocaloric materials is a key parameter for evaluating performance in a magnetic refrigerator. In an ideal refrigeration cycle, RCP is the amount of heat transferred between hot and cold reservoirs, and can be estimated using the following equation [34]:

(2) $$RCP={∆S}_{M}^{max}\times {\delta T}_{FWHM}$$

where ${∆S}_{M}^{max}$ is the maximum magnetic entropy change value and ${\delta T}_{FWHM}$ is the full width at half maximum of the magnetic entropy curve. Figure 5b shows the RCP values for HoB_2_ and Ho_0.93_Nb_0.07_B_2_, which increase linearly with the applied magnetic field. Ho_0.93_Nb_0.07_B_2_ shows lower RCP values compared to HoB_2_ at high field strengths (i.e., >3 T). The RCP values for other SOPT Ho-based compounds are also shown in Figure 5b.

## 4. Discussion

Liquefaction is important for gas storage and transportation, as is evident for the natural gas industry [35] and for specific existing uses of hydrogen [5]. The broadened utilization of liquid hydrogen for “hard-to-abate” industry sectors [36], as well as for transportation [36], may be rapidly effected by more compact and efficient technologies such as MR [8]. Continuous cooling using magnetocaloric materials requires both rapid variation in magnetic fields [37] and the use of magnetic material as a regenerator in an Active Magnetic Regenerative Refrigerator (AMRR) [8,18]. A large entropy change for magnetocaloric materials is preferably obtained at magnetic fields above 2 T [16], most effectively deployed with superconducting magnets [37]. In general, the value for magnetic enthalpy, |ΔS_M_|, increases with an increased applied magnetic field [38], as shown in Figure 5a for both Ho diboride compounds up to 10 T and for other Ho compounds up to 5 T [21,22,32].

As shown by de Castro et al., [22] the magnetic entropy change for HoB_2_ is at a maximum value of 40.1 J. kg^−^^1^K^−^^1^ near the Curie temperature (T_C_~15 K) for a magnetic field change of 5 T [19,38,39,40,41]. Because the Curie temperature for HoB_2_ is close to the temperature for liquid hydrogen (20.3 K), this compound is a promising candidate for AMRRs [22,38]. However, the maximum change in entropy, ΔS_M_, for HoB_2_, decreases with the increasing temperature for the same applied field, and so is of optimum practical use at, or near, 15 K. Hence, a range of elements have been substituted into HoB_2_ [21,32,39] in order to either (i) increase the T_C_ and/or to (ii) increase ΔS_M_ across a broad temperature range.

Iwasaki et al. [39] show that, in the absence of a solid solution between end-member alloys (e.g., HoB_2_ and HoAl_2_), the MCE is only due to that of the dominant magnetocaloric material. As a result, it is important to establish the solubility limit(s) of potential substituents of HoB_2_ or similar alloys. For other substituted alloys, such as Ho_1−x_Dy_x_B_2_ [32], T_C_ increases with increased Dy substitution up to x = 1 for an applied field of 5 T. However, with increased T_C_, the magnitude of |ΔS_M_| decreases, although the temperature range of the |ΔS_M_| curve increases [32]. Similarly, for Ho_1-x_Gd_x_B_2_ alloys (for 0 < x < 0.4), an applied field of 5 T results in an increase in T_C_ and broadens the |ΔS_M_| curve(s) without the magnetic hysteresis effects of the Dy analogue [21]. For the Gd-substituted series, the T_C_ increased to between 17 K and 30 K [21]. This increase in T_C_ offers the potential to deliver a relatively high refrigerant capacity across a wide temperature range (e.g., from 15 K to 30 K or higher) with HoB_2_ and appropriate stoichiometric substitutions of soluble elements.

The phase field for Ho with B shows that HoB_2_ forms at a peritectic temperature of 2200 °C due to the decomposition of solid HoB_4_ and a Ho-rich liquid, with complete formation at 2350 °C [42]. Noting the relatively low purity of Ho starting material at 95% (Supplemental Table S1), we suggest that the presence of impurities in the final product may be reduced with higher quality Ho. We have considered the potential for impurity phases, such as Ho_2_O_3_ and HoB_4_, to affect the magnetocaloric properties of HoB_2_ and Nb-substituted HoB_2_. For HoB_4_, the presence of antiferromagnetic transitions at 7.1 K and 5.7 K [43,44] are a proxy indicator for effect(s) on the magnetocaloric properties of HoB_2_ samples. Similarly, an antiferromagnetic transition occurs for amorphous and crystalline Ho_2_O_3_ at 2.1 K and 5.2 K, respectively [45,46]. As shown in Figure 3a,b, such anomalies are not observed for HoB_2_ and Ho_0.93_Nb_0_._07_B_2_. Therefore, we suggest that the minor amounts of HoB_4_ and Ho_2_O_3_ have a limited or no effect on the magnetic transition temperature of these arc-melted samples.

Both Ho-boride samples show a pronounced peak at or near the Curie temperature, T_C_. The T_C_ increase to 17.5 K for Ho_0.93_Nb_0.07_B_2_ is due to successful substitution of Nb into the HoB_2_ structure. A second magnetic transition marked by T^*^ at 11 K is observed for both HoB_2_ and Nb-substituted HoB_2_. The origin of T^*^ is likely due to a spin reorientation mechanism, as identified for Dy- and Gd-substituted HoB_2_ [21,32]. The addition of a spherical S^7/2^ Gd^3+^ moment in HoB_2_ induces an enhancement in the Curie temperature, a reduction in the peak value of ΔS_M_, and a broadening of the ΔS_M_ curves [21]. The values for Ho_0.93_Nb_0_._07_B_2_ show a similar trend, with an increase in T_C_ and reduction of ΔS_M_ for all applied fields up to 10 T. We suggest that the increased substitution of Nb into HoB_2_ will further increase T_C_, with a consequent increase in the temperature range at which ΔS_M_ is viable for effective MR.

The relative reduction in RCP for Ho_0.93_Nb_0_._07_B_2_ compared with HoB_2_ can also be attributed to the substitution of Nb for Ho, which alters magnetic interactions and the structural ordering within the material. Nb substitution weakens the overall magnetic moment and decreases MCE efficiency, thereby reducing the RCP. Despite the lower RCP, the linear trend in Figure 5b suggests that both materials maintain a predictable response to increasing fields, suggesting that Nb-substituted HoB_2_ compounds are candidates for further exploration in field-dependent cooling applications.

Table 3 summarizes the magnetocaloric properties of HoB_2_ and Ho_0.93_Nb_0.07_B_2_ from this study, as well as for other SOPT Ho compounds [8,21]. The maximum ΔS_M_ value for HoB_2_ in this work under an applied field of 5 T (34.3 Jkg^−^^1^K^−^^1^) is comparable with the value of 40.1 Jkg^−^^1^K^−^^1^ for arc-melted samples reported by de Castro et al. [22]. The ΔS_M_ value reported for gas-atomized particles of HoB_2_ near the T_C_ of 15 K is also 40.1 Jkg^−^^1^K^−^^1^ [47], and slightly lower in the data reported by Yamomoto et al. [41]. In these cases, XRD data show that the HoB_2_ samples contain minimal or no impurities [22,41,47], unlike those noted in Table 1 for HoB_2_ in this work and for Ho_0.93_Nb_0_._07_B_2_.

For HoB_2_ produced in this study and shown in Table 1, impurities account for ~9% of the final ingot and thus, a lower weight fraction of HoB_2_. Similarly, for Ho_0.93_Nb_0_._07_B_2_, the reduction in weight fraction of other Ho compounds is ~25%. Adjusting the weight-dependent values for both samples suggests that at an applied field of 5 T, the maximum ΔS_M_ for HoB_2_ and Ho_0.93_Nb_0_._07_B_2_ from this study would be ~38 Jkg^−^^1^K^−^^1^ and ~35 Jkg^−^^1^K^−^^1^, respectively. This approach is consistent with estimates of ΔS_M_ by Iwasaki et al. [39] when evaluating the impact of Al substitution into HoB_2_. For an applied field of 10 T, the weight-adjusted values of a maximum ΔS_M_ are ~52 Jkg^−^^1^K^−^^1^ and ~49 Jkg^−^^1^K^−^^1^ for HoB_2_ and Ho_0.93_Nb_0_._07_B_2_, respectively.

For both examples of HoB_2_ in Table 3, the RCP values are similar at 5 T and suggest that marginal differences in magnetic parameters (e.g., T_C_ and ΔS_M_) and/or a modest level of impurities may enable a comparable refrigeration capacity in an operating system. In practice, the data in Table 1 and Table 3 imply that low levels of impurities are unlikely to substantially affect ΔS_M_ at high applied fields (i.e., > 2 T). This implication is consistent with a detailed study on the presence of non-stoichiometric phases formed by inductive melting gas atomization [41]. The work by Yamomoto et al. [41] showed that the presence of up to 20 wt. % impurity phases has minimal effect on the physical properties of HoB_2_ particles, while retaining a value for ΔS_M_ well above 30 Jkg^−^^1^K^−^^1^ at 5 T. By comparing the maximum values of ΔS_M_ for HoB_2_ at 5 T from earlier works [22,30,41,47] and this study, we estimate an average value of 39.1 (±1.5) Jkg^−^^1^K^−^^1^, suggesting that sample preparation for HoB_2_ may have a limited influence on magnetic properties. Nevertheless, for HoNi synthesis, Rajivghandi et al. [48] have shown a difference of 8 K in the T_C_ values between melt-spun and arc-melted samples. The values for HoNi in Table 3 are for an arc-melted sample [48].

The identification of Nb solubility in HoB_2_ suggests that higher proportions of Nb, or other Group 5 elements, may enable a family of Ho_1-x_M_x_B_2_ compounds (where M = Gd, Nb) suited to hydrogen liquefaction. With a combination of SOPT compounds, as noted above, or of similar analogues, an AMRR system based only on Ho compounds may be a realizable goal. SOPT magnetic materials, which include the compounds listed in Table 3, and now including Ho_0.93_Nb_0.07_B_2_,_,_ are without thermal hysteresis, and offer the capacity for reversibility and mechanical stability in a system undergoing cyclic performance [19]. While temperatures from 77 to 20 K are not completely covered by the combination of compounds in Table 3, there is potential to extend the T_C_ range from 15 to 36 K. The use of Ho compounds with additional element substitutions (e.g., HoCo_1.8_Ni_0.15_Al_0.05_), as exemplified by Tang et al. [19], and/or including Group 5 elements, show promise.

Superconducting magnets are necessary to achieve and maintain a higher field strength compared to magnetic field(s) from permanent magnets. This distinction is crucial for some practical applications, particularly for MR at cryogenic temperatures below 113 K [5], and as shown in this and previous works [17,19,21,22]. The reduction in ∆S_M_ with Nb substitution in HoB_2_ suggests a trade-off between increasing the T_C_ and maintaining a high magnetocaloric effect, as noted by others [21]. Table 3 shows that for SOPT polycrystalline Ho compounds, both the ∆S_M_ and RCP values increase with an increased applied field up to 10 T. These data also suggest that despite a drop in the ΔS_M_ value, with higher T_C_ for a particular compound, the range of values for δT_FWHM_ available at higher magnetic fields (i.e., >5 T) provides an effective RCP using SOPT Ho-based magnetocaloric materials.

## 5. Conclusions

We have synthesized the alloy of composition Ho_0.93_Nb_0.07_B_2_ using arc melting, and compared its magnetic and microstructural properties with HoB_2_ prepared by the same technique. Substitution of Nb in HoB_2_ results in an increased T_C_ and a decrease in the ΔS_M_ and RCP in response to changes in the magnetic field up to 10 T. Arrott plots confirm a second-order phase transition in these Ho compounds. The maximum ΔS_M_ values for HoB_2_ and Ho_0.93_Nb_0_._07_B_2_ measured from this study are 34.3 Jkg^−^^1^K^−^^1^ and 26.4 Jkg^−^^1^K^−^^1^ at 5 T and 46.8 Jkg^−^^1^K^−^^1^ and 38.2 Jkg^−^^1^K^−^^1^ at 10 T, respectively. These polycrystalline samples contained other impurity phases, such as unreacted Ho and Nb, Ho_2_O_3_, and HoB_4_. Adjusting for impurity phases in both samples indicates that higher values of ΔS_M_ and RCP may be achievable. This work adds another compound, Ho_0.93_Nb_0.07_B_2_, to the metal diboride class of magnetocaloric materials with potential for additional and commensurate properties suited to magnetic refrigeration.

## Figures and Tables

**Table 1:** Synthesis conditions and products.

Sample ID	Starting Materials	Reactant Ratio	Products
HoB_2_	Ho:B	1:2	HoB_2_ (92.1%), Ho_2_O_3_ (4.0%), Ho (3.9%)
(Ho,Nb)B_2_	Ho:Nb:B	0.9:0.1:2	HoB_2_ (72.1%), HoB_4_ (9.2%), Ho_2_O_3_ (4.6%), Ho (4.6%), HoB_12_ (3.8%), NbB_2_ (3.2%)

**Table 2:** Cell parameters for selected metal diborides.

Sample	a (Å)	c (Å)	Reference
HoB_2_	3.28296(2)	3.81445(0.4)	This work
Ho_1−x_Nb_x_B_2_	3.26801(0.6)	3.78415(0.1)	This work
HoB_2_	3.2835(4)	3.8186(14)	PDF# 04-003-0232
* NbB_2_	3.1049(3)	3.2990(2)	PDF# 04-014-5978

Estimated errors for all samples are in parentheses; * based on data from ref [25].

**Table 3:** Magnetocaloric properties for selected SOPT Ho compounds.

Alloy	T_C_ (K)	${∆S}_{M}$ (Jkg^−1^K^−1^)		RCP (Jkg^−1^)		Reference
		At 5 T	At 10 T	At 5 T	At 10 T	
HoB_2_	15	39.2	-	706 *	-	[41]
HoB_2_	15.8	34.3	46.8	720	1474	This work ^#^
Ho_0.93_Nb_0.07_B_2_	17.5	26.4	38.2	673	1337	This work ^#^
Ho_0.9_Gd_0.1_B_2_	19	34.6	-	833	-	[21]
Ho_0.6_Gd_0.4_B_2_	30	20.2	-	889	-	[21]
HoAl_2_	29	21.5	30 *	688 *	1350 *	[17]
HoNi	36	17.4	~26 *	750	1222 *	[48]
HoN	18	28.2	-	846 *	-	[49]

* values estimated from δT_FWHM_ data in reference; ^#^ without weight-dependent correction (see below).

## Data Availability

The original contributions presented in this study are included in the article/supplementary material. Further inquiries can be directed to the corresponding author.

## Supplementary Materials

The following supporting information can be downloaded at: https://www.mdpi.com/article/10.3390/ma18040866/s1. Table S1: ICP-OES Analysis of trace/minor elements in starting Ho powder; Figure S1: Powder XRD patterns for 25° < 2θ < 50° for HoB_2_ and Ho_0.93_Nb_0.07_B_2_; Figure S2: Powder XRD patterns for 60° < 2θ < 83° for HoB_2_ and Ho_0.93_Nb_0.07_B_2_; Figure S3: Plot showing unit cell parameters for HoB_2_, Ho_0.93_Nb_0.07_B_2_, and NbB_2_.
