# Supplementary material for: Magnetocaloric Properties and Microstructures of HoB2 and Nb-Substituted HoB2
Source: Materials (Basel). 2025 Feb 17;18(4):866. doi: 10.3390/ma18040866 (PMC11857285; doi:10.3390/ma18040866)
Supplement: Supplementary file 1 [file materials-18-00866-s001.zip › materials-3464618-supplementary.pdf]

Supplemental Materials

Magnetocaloric Properties and Microstructures of HoB_2 and Nb-Substituted HoB_2

Mahboobeh Shahbazi[1,2], Ali Dehghan Manshadi[3], Kiran Shinde[4], Ian Mackinnon[2,5,*]

[1] Centre for Materials Science and School of Chemistry and Physics, Queensland University of Technology,
Brisbane, QLD 4001, Australia; mahboobeh.shahbazi@qut.edu.au
[2] Centre for Clean Energy Technologies and Practices, Queensland University of Technology, Brisbane, QLD 4001, Australia
[3] School of Mechanical and Mining Engineering, The University of Queensland, Brisbane, QLD 4072, Australia; aliuow@gmail.com
[4] Department of Nanotechnology and Advanced Materials Engineering, Sejong University, Seoul 05006, Republic of Korea; yourkirans@gmail.com
[5] School of Earth and Atmospheric Sciences, Queensland University of Technology, Brisbane, QLD 4001, Australia
* Correspondence: ian.mackinnon@qut.edu.au

Table S1: ICP-OES Analysis of trace/minor elements in starting Ho powder

**Analyte**	**Ho bulk (mg/kg)**
11 B	8.136
24 Mg	<4.294
27 Al	2.129
40 Ca	85.221
47 Ti	<0.672
55 Mn	3.56
56 Fe	19.005
60 Ni	74.696
63 Cu	157.951
89 Y	20.953
125 Nb	49.22
139 La	0.517
140 Ce	0.339
141 Pr	0.105
146 Nd	0.616
147 Sm	0.541
157 Gd	0.129
159 Tb	0.129
163 Dy	722.425
166 Er	536.749
169 Tm	51.593
172 Yb	0.052
175 Lu	25.829
213 Ta	3699.515
214 W	164.924
**Totals (mg/kg)**	5624.334

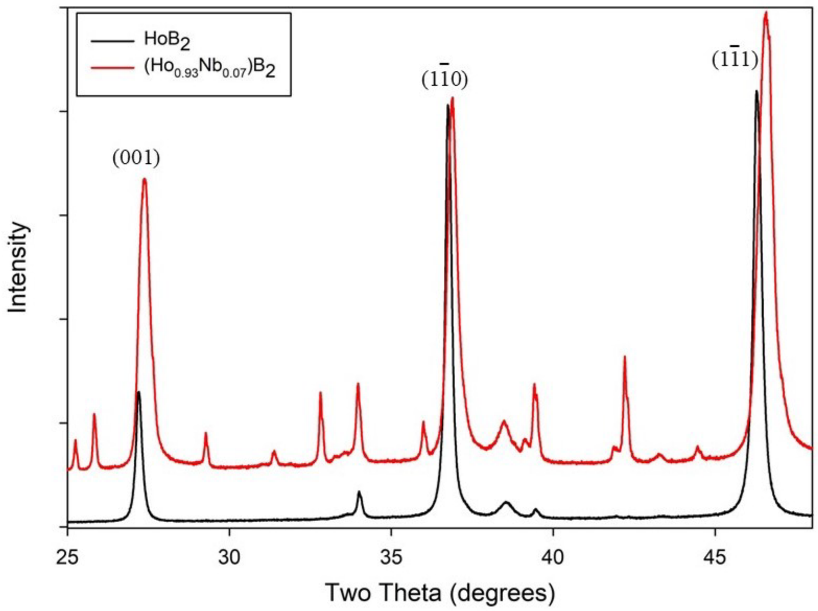

Figure S1: Powder XRD patterns for 25º < 2θ < 50º for HoB_2 (black) and $Ho_{0.93}Nb_{0.07}B_2$ (red) produced by arc melting as listed in Table 1. Peaks for the HoB_2 structure are indexed. Note the shift of peaks for $Ho_{0.93}Nb_{0.07}B_2$ to higher values of 2θ compared to HoB_2.

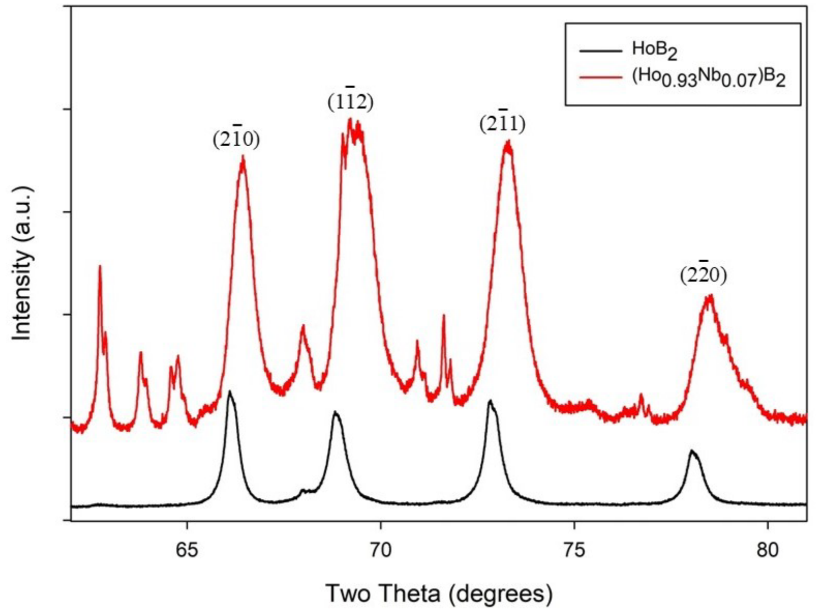

Figure S2: Powder XRD patterns for 60º < 2θ < 83º for HoB_2 (black) and $Ho_{0.93}Nb_{0.07}B_2$ (red) produced by arc melting as listed in Table 1. Peaks for the HoB_2 structure are indexed. Note the broadening of peaks for $Ho_{0.93}Nb_{0.07}B_2$ and shift to higher values of 2θ compared to HoB_2.

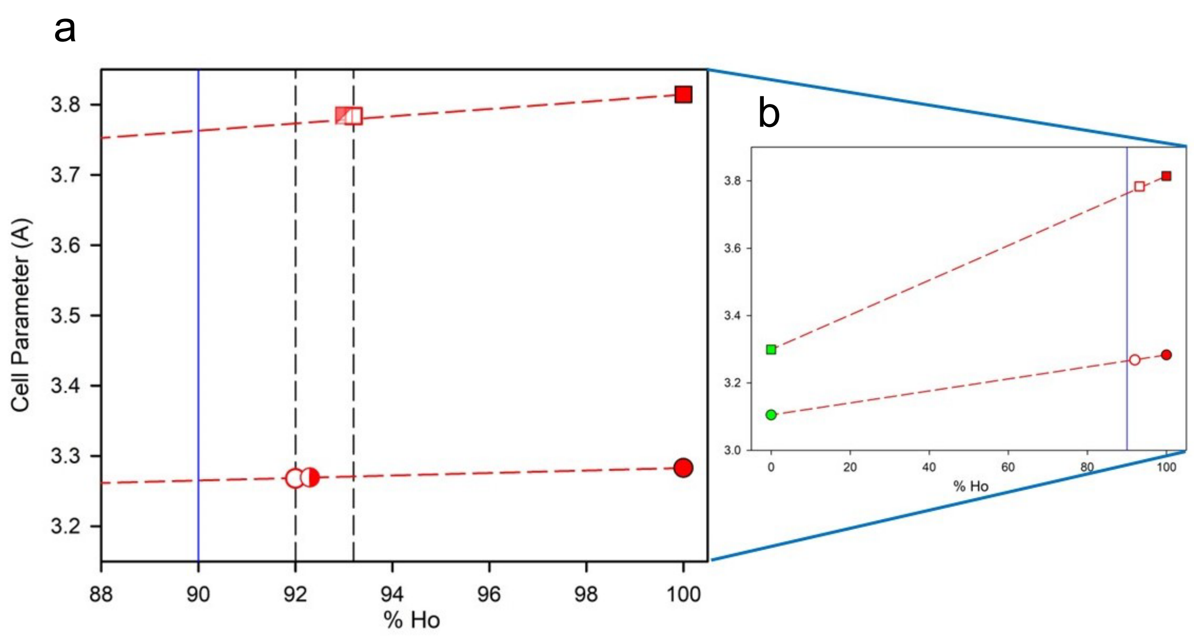

<u>Figure S3:</u> Plot showing unit cell parameters, *a* (filled circles) and *c* (filled squares) for HoB_2 (red) and NbB_2 (green) in Figure S3(b). The vertical blue line at 90% Ho content is at the nominal substitution of Nb based on initial synthesis mixture listed in Table 1. Open symbols show values for the refined unit cell determined for the $(Ho_{1-x}Nb_x)B_2$ arc melted product. Nb substitution is between 8.0 % (*a* axis) and 6.8 % (*c* axis) defined by the dotted vertical lines in Figure S3(a); half-filled symbols represent values for crystallite size corrections derived from SEM images shown in Figure 2. These crystallite size corrections are within the substitutional range identified in Figure S3(a) (*e.g.* 7.75% and 7.0% Nb, respectively). We conclude from Figure S3 that Nb is soluble in HoB_2.
